# Mutation analysis and clinical profile of South African patients with Neurofibromatosis type 1 (NF1) phenotype

**DOI:** 10.3389/fgene.2024.1331278

**Published:** 2024-03-26

**Authors:** Maria Mabyalwa Mudau, Bronwyn Dillon, Clarice Smal, Candice Feben, Engela Honey, Nadia Carstens, Amanda Krause

**Affiliations:** ^1^ Division of Human Genetics, National Health Laboratory Service and School of Pathology, Faculty of Health Sciences, University of the Witwatersrand, Johannesburg, South Africa; ^2^ Department of Biochemistry, Genetics and Microbiology, Faculty of Natural and Agricultural Sciences, University of Pretoria, Pretoria, South Africa; ^3^ Genomics Platform, South African Medical Research Council, Cape Town, South Africa

**Keywords:** Neurofibramotosis type 1, single nucleotide variant (SNV), copy number variants (CNV), next generating sequencing, MLPA (multiplex ligation-dependent probe amplification)

## Abstract

Neurofibromatosis type 1 (NF1) is an autosomal dominant genetic condition with complete age-dependent penetrance, variable expressivity and a global prevalence of ∼1/3,000. It is characteriszed by numerous café-au-lait macules, skin freckling in the inguinal or axillary regions, Lisch nodules of the iris, optic gliomas, neurofibromas, and tumour predisposition. The diagnostic testing strategy for NF1 includes testing for DNA single nucleotide variants (SNVs), copy number variants (CNVs) as well as RNA analysis for deep intronic and splice variants, which can cumulatively identify the causative variant in 95% of patients. In the present study, NF1 patients were screened using a next-generation sequencing (NGS) assay targeting *NF1* exons and intron/exon boundaries for SNV and *NF1* multiple ligation-dependent probe amplification (MLPA) analysis for CNV detection. Twenty-six unrelated Southern African patients clinically suspected of having NF1, based on the clinical diagnostic criteria developed by the National Institute of Health (NIH), were included in the current study. A detection rate of 58% (15/26) was obtained, with SNVs identified in 80% (12/15) using a targeted gene panel and *NF1* gene deletion in 20% (3/15) identified using MLPA. Ten patients (38%) had no variants identified, although they met NF1 diagnostic criteria. One VUS was identified in this study in a patient that met NF1 diagnostic criteria, however there was no sufficient information to classify variant as pathogenic. The clinical features of Southern African patients with NF1 are similar to that of the known NF1 phenotype, with the exception of a lower frequency of plexiform neurofibromas and a higher frequency of developmental/intellectual disability compared to other cohorts. This is the first clinical and molecular characterisation of a Southern African ancestry NF1 cohort using both next-generation sequencing and MLPA analysis. A significant number of patients remained without a diagnosis following DNA-level testing. The current study offers a potential molecular testing strategy for our low resource environment that could benefit a significant proportion of patients who previously only received a clinical diagnosis without molecular confirmation.

## Introduction

Neurofibromatosis type 1 (NF1; OMIM#162200) is an autosomal dominant genetic condition with complete penetrance and variable expressivity. It is a relatively common genetic disorder with a global prevalence of 1/2,500–1/3,000 live births. This neurocutaneous condition is characterized by variable expression with features including, but not limited to, cutaneous manifestations (e.g., multiple café-au-lait macules, and inguinal/axillary skin freckling), ocular findings (e.g., Lisch nodules of the iris and optic gliomas), neurological features (e.g., intellectual and learning disabilities, behavioural problems, and seizures), and a predisposition to the development of tumours (e.g., neurofibromas, plexiform neurofibromas, malignant peripheral nerve sheath tumours, brain tumours, breast cancer, and haematologic malignancies) (Cimino and Gutmann, 2018; Mao et al., 2018; Kang and Lee, 2019; Jafry and Sidbury, 2020). Individuals affected with NF1 typically have a below-average length/height and an above-average head circumference (Clementi et al., 1999; Soucy et al., 2013). Despite the variable clinical expressivity of NF1, several key criteria developed by the National Institutes of Health (NIH) (NIH, 1988) and revised by Legius et al. (2021) allow for the clinical diagnosis of this condition. Molecular genetic analysis may be required in certain instances such as for those individuals not meeting NIH diagnostic criteria, in the prenatal or preclinical genetic testing setting, genotype-phenotype correlation as well as in monitoring/surveillance of the affected individuals (Anderson and Gutmann, 2015; Legius et al., 2021).

NF1 was the first disorder identified to implicate a gene involved in the RAS/mitogen-activated protein kinase (RAS-MAPK) pathway, and is thus considered a RASopathy (Viskochil et al., 1990). It is caused by loss of function pathogenic variants in the neurofibromin 1 (*NF1*) tumor suppressor gene, located on the long arm of chromosome 17 (17q11.2). The *NF1* gene encodes neurofibromin, a guanosine triphosphatase–activating protein that inhibits RAS activity. Neurofibromin catalyses RAS GTPase activity, thereby negatively regulating the RAS/MAPK signalling pathway. Pathogenic loss of function variants in the NF1 gene thus leads to the hyperactivation of RAS/MAPK signalling (Jafry and Sidbury, 2020) resulting in abnormal cell growth, differentiation and proliferation and the subsequent phenotype of NF1 (Friedman, 1993a; Mao et al., 2018). The *NF1* gene is one of the largest known genes with a genomic size of 282 kb, consisting of 57 constitutive exons and three alternatively spliced exons without any obvious hotspot regions. Over 2600 *NF1* pathogenic variants have been reported in the Human Gene Mutation Database (HGMD) and ClinVar disease variant databases to date (Stenson et al., 2003; Landrum et al., 2018).

Approximately 50% of NF1 cases are familial and the other 50% arise *de novo* (Anderson and Gutmann, 2015). Patients with NF1 caused by large deletions such as whole-gene deletions (termed *NF1* microdeletions) present with somatic overgrowth, dysmorphism, an earlier and heavier burden of cutaneous and plexiform neurofibromas, an increased risk of malignant peripheral nerve sheath tumour development, and more severe neurocognitive delay compared to individuals with single nucleotide variants (Cnossen et al., 1997; Mautner et al., 2010).

The diagnostic testing strategies for NF1 include a series of investigations, which can cumulatively identify the causative variant in 95% of clinically affected individuals. Next-generation sequencing (NGS) technology using a targeted gene panel, targeting all exons of the *NF1* gene, is able to identify 60%–90% of single nucleotide variants (SNVs) (Cimino and Gutmann, 2018). RNA analysis is reported to identify splice variants in 22%–30% of patients (Evans et al., 2016). Copy number variants (CNVs) have also been reported to cause NF1 in 5%–10% of patients (Imbard et al., 2015) and are detected using techniques such as microarray and multiplex-ligation dependent probe amplification (MLPA). These molecular testing strategies are used widely in most diagnostic laboratories for the molecular diagnosis of NF1, however somatic mosaicism may occur and make variant detection challenging (Messiaen and Xie, 2012).

Legius syndrome (also known as NF1-like syndrome) has clinical features that overlap with those observed in NF1 patients, such as multiple café-au-lait macules, freckling and learning difficulties. The molecular diagnosis of Legius syndrome is confirmed by the identification of a heterozygous pathogenic variant in the *SPRED1* gene. Thus when analysing NF1 patients, it is recommended to screen both the *NF1* and *SPRED1* genes for variants (Brems et al., 2012; Legius et al., 2021).

In the African context, NF1 studies have focused on the characterisation of the clinical phenotype in probands and their relatives. A study by Sendrasoa et al. (2022) in Madagascar that included 28 patients with NF1 indicated clinical variability among the individuals as well as variable inheritance patterns, i.e., ∼61% sporadic and ∼39% *de novo* (Sendrasoa et al., 2022). Older studies in South Africa (Ramanjam et al., 2006) and Nigeria (Odebode et al., 2005) also focused on the clinical features of the NF1 cohorts in their various settings. These studies reported similar common clinical features such as café au lait spots, and benign neurofibromas, with the Nigerian study reporting a higher ratio of males to females (3:2) in their cohort and the highest density of neurofibromas covering the extremities (Odebode et al., 2005). The South African study which included 48 young patients (4–12 years) with a similar ratio of males and females reported learning and behavioural problems in ∼70% of the patients and this reinforced the importance of neuropsychology assessments in all children with reported school problems as well as enabling formal developmental assessments and planning of specific educational placement to optimize learning (Ramanjam et al., 2006). A study in Egypt by Abdel-Aziz et al. (2021) on the mutational spectrum of the *NF1* gene in patients presenting with NF1, achieved a 96% detection rate with 24 of the 25 patients meeting the NIH diagnostic criteria for NF1 (Abdel-Aziz et al., 2021).

In the current study, patients of African ancestry with a suspected clinical diagnosis of NF1 were screened for SNVs and CNVs using a multi-disease targeted NGS panel and MLPA analysis of the *NF1* and *SPRED1* genes. There is currently no information available in the literature about the molecular aetiology in South African patients of African ancestry with NF1. A study performed in our laboratory demonstrated that NGS targeted gene panels are a cost-effective strategy that can be employed for the molecular diagnosis of a wide range of monogenic disorders in a low resource setting (Mudau et al., 2023). The current study aims to develop a molecular testing strategy that would identify the majority of *NF1* variants and would be feasible for molecular diagnostic laboratories to implement, in low resource settings.

## Methods

### Participants

Twenty-six unrelated patients of African ancestry clinically suspected to have NF1 were recruited from Genetic clinics across the Gauteng province of South Africa and consented to participate in the RASopathy study of the Division of Human Genetics, National Health Laboratory Service and the University of the Witwatersrand. The diagnosis of NF1 was based on whether the patients presented with clinical features meeting the diagnostic criteria for NF1 initially developed by the NIH (Legius et al., 2021). The NIH diagnostic criteria for NF1 is described in A and B as follows; A:The diagnostic criteria for NF1 can be met in an individual with two or more of the following are present: Six or more café-au-lait macules over 5 mm in greatest diameter in prepubertal individuals and over 15 mm in greatest diameter in postpubertal individuals; Freckling in the axillary or inguinal regiona; two or more neurofibromas of any type or one plexiform neurofibroma; optic pathway glioma; two or more iris Lisch nodules and a heterozygous pathogenic *NF1* variant with a variant allele fraction of 50% in apparently normal tissue such as white blood cells.

B: A child of a parent who meets the diagnostic criteria specified in A merits a diagnosis of NF1 if one or more of the criteria in A are present.

Two patients (patient 9 and 11) did not fully meet the NIH clinical diagnostic criteria however; they were included in the study on the basis of strong supporting features of NF1; in addition patient 11 and patient 8 had features suggestive overlapping Noonan syndrome and NF1 (see Supplementary Table S1). The 26 patients underwent a clinical examination, including anthropometric measurements. A retrospective, descriptive review was performed on clinical data obtained from the 26 patients for whom data were available. Comprehensive clinical notes were not available for all patients and the number of patients with available data varied across the characteristic categories. Fisher’s exact test was used to calculate the *p*-value from a 2 × 2 contingency table of categorical variables. Differences in means were considered statistically significant with *p* values <0.05.

Ethics clearance was obtained from the University of the Witwatersrand Human Research Ethics Committee (HREC) (Medical) for the RASopathy studies (protocol numbers M170163 and M180506) and the University of Pretoria Research Ethics Committee (80/2018). Blood samples were obtained from the patients and DNA was extracted using a modified version of the salting-out method (Miller et al., 1988). DNA concentrations and quality were determined using the NanoDrop Spectrophotometer and Qubit fluorometer using dsDNA broad-range and high-sensitivity assay kits (Invitrogen by Thermo-Fisher Scientific, Inc.).

### Molecular studies

The *NF1* gene was sequenced using an NGS approach targeting only exons and 10bp of the exon/intron boundaries for each exon. Agilent SureSelect^QXT^ and IonTorrent Ion Ampliseq capture assays to target the exons and exon/intron boundaries of the *NF1* (NM_001042492.3)*, SPRED1* (NM_152594.3). Other RASopathy genes were also sequenced (Mudau et al., 2023), these included; *A2ML1* (NM_144670.6), BRAF (NM_004333.4), *CBL* (NM_005188.4), *HRAS* (NM_005343.4), *KRAS* (NM_033360.4), *LZTR1* (NM_006767.3), *MAP2K1* (NM_002755.3), *MAP2K2* (NM_030662.3), *NF2* (NM_000268.4), *NRAS* (NM_002524.3), *PTPN11* (NM_0002834.3), *RAF1* (NM_002880.3), *RASA1* (NM_002890.3), *RASA2* (NM_006506.5), *RIT1* (NM_006912.6), *RRAS* (NM_006270.5), *SHOC2* (NM_007373.4), *SOS1* (NM_005633.4), *SOS2* (NM_006939.4). The two targeted gene panels utilised in the current study were custom designed using Agilent SureDesign Software (Agilent Technologies,United States) and ThermoFisher Ion AmpliSeq Designer (ThermoFisher Scientific, United States). Sequencing was done using the MiSeq (Illumina, CA, United States) and Ion S5 (ThermoFisher Scientific, United States) sequencing platforms, respectively, at The University of the Witwatersrand, Johannesburg, South Africa. Both these platforms have built-in parameters that perform basecalling (based on >90% of sequence read having quality score of Q30), sequence alignment to the h19 human reference genome as well as variant calling. These processes allowed for output of raw sequence format (FASTQ), binary alignment map (BAM) and variant call format (VCF) files. The VCF file obtained from each sample was annotated using Ensembl Variant Effect Predictor (VEP) (McLaren et al., 2016). Variants with a minor allele frequency above 0.025 in the Ensembl VEP population databases were excluded from further analysis. The minor allele frequency cut-off was informed by the American College of Medical Genetics and Genomics and Association for Molecular Pathology (ACMG-AMP) guidelines (Richards et al., 2015) and the ClinGen expert panel for RASopathies (Gelb et al., 2018). All disease-causing variants were evaluated for sequencing depth coverage (>30x) and quality information (allele ratio of ∼50% between the reference and the patient’ variant site with no observed allele/strand bias) by manual visualisation on the Integrative Genomics Viewer (IGV) (Robinson et al., 2017) using BAM files obtained per patient. Clinically significant variants were subsequently classified using the ACMG-AMP and the ClinGen RASopathy expert panel guidelines to identify putative disease-causing variants. SpliceAI tool was used as a plugin on the VEP software to assess if there were any high impact splice site variants from exon/intron boundaries. SpliceAI predicts splicing defects caused by DNA variations by giving delta scores that could be used to assess the level of likely splicing effect (de Sainte Agathe et al., 2023).

### MLPA analysis

MLPA was used to detect CNVs in patients clinically diagnosed with NF1, but in whom no pathogenic SNVs were detected using the NGS panels. Commercially available SALSA P081 NF1 (version D1), P082 NF1 (version C2) and P122 NF1 (version D2) area MLPA probe mixes were used (MRC Holland, Netherlands). The P081 and P082 mixes collectively contain at least one probe for each exon of the *NF1* gene and are able to detect deletions and duplications in the *NF1* gene. The P122 probe mix detects deletions and duplications in the region surrounding the *NF1* gene including the *ADAP2, ASPA, ATAD5, BLMH, CPD, CRLF3, LRRC37B, MYO1D, NF1, PMP22, PSMD11, RNF135, SUZ12, SUZ12P1, TRAF4, UTP6, ZNF207, UTP6* and *ZNF207* genes. SALSA MLPA EK1 Reagent kit (FAM labelled) (MRC Holland, Netherlands) was also used as per the manufacturer’s instructions. Capillary electrophoresis was performed using the GeneScan 500 LIZ size standard and the Applied Biosystems 3500xl Genetic Analyzer (ThermoFisher Scientific, United States). MLPA fragments were analysed using the Coffalyser.Net software (MRC Holland).

## Results

The study consisted of 26 unrelated Southern African patients of various ethnolinguistic groups. Ten (38%) were female and 16 (62%) male. The median age was ∼12 years old (range 6 months–42 years). An NF1-causing variant was identified in 15/26 (58%) patients, a VUS was identifed in 1/26 patient (4%) (patient 8), and 10/26 (38%) did not have any clinically significant SNV or CNV identified. Twelve patients, 12/26 (46%) of the study group had pathogenic/likely pathogenic SNVs identified using the NGS gene panel. Three patients, 3/26 (12%) had CNVs identified using MLPA analysis. The molecular findings are outlined in Table 1. In the present study, the 15 patients with a pathogenic/likely pathogenicvariant identified are referred to as the positive cohort in the discussion and the 10 patients with no pathogenic variant identified as the negative cohort. The main clinical characteristics of the positive and negative cohorts are outlined and compared in Table 2. More detailed clinical information can be found in Supplementary Tables S1, S2. In the positive cohort, 4/15 (27%) of patients, for whom the information was available, had a documented first degree relative with clinically-diagnosed NF1. Detailed phenotypic information was not available for these relatives.

**TABLE 1:** Molecular findings of the 15 patients with heterozygous NF1-causing variant and one VUS identified in Patient 8 classified using ACMG-AMP guidelines. The variants were described using HGVS (SNVs) and ISCN (CNVs) nomenclature and *NF1* gene transcript NM_001042492.3

Patient	c.notation	p.notation	Type	ACMG-AMP codes applied (Richards et al., 2015)	Classification	ClinVar ID
1	c.1658A>G	p.His553Arg	Missense	PP3,PP4,PP5,PM2,PM5^b^ (ClinVar-1777378) and (ClinVar-1454369)	Pathogenic	420076
2	c.6772C>T	p.Arg2258Ter	Nonsense	PP5,PM2,PVS1	Pathogenic	230389
3	c.496_497del	p.Val166Leufs	Frame-shift	PP4,PP5,PM2,PVS1	Pathogenic	431562
4	c.3721C>T	p.Arg1241Ter	Nonsense	PP4,PP5,PM2,PVS1	Pathogenic	361
5	c.569T>G	p.Leu190Ter	Nonsense	PP4,PP5,PM2,PVS1	Pathogenic	431974
6	c.5267_5268del	p.Lys1756Serfs	Frame-shift	PP4,PP5,PM2,PVS1	Pathogenic	1072614
7	c.27G>A	p.Trp9Ter	Nonsense	PP4,PP5,PM2,PVS1	Pathogenic	1459748
8^a^	c.1885G>C	p.Gly629Arg	Missense	PP4,PM2, PS1 (downgraded to moderate)	VUS	1410579
9	:c.5991G>A	p.Trp1997Ter	Nonsense	PP5,PM2,PVS1	Pathogenic	233869
10	c.2540T>C	p.Leu847Pro	Missense	PP3,PP4,PP5,PM2,PM5^b^ (Clinvar-573019),PS3^c^ (PMID:16513807)	Pathogenic	68323
11^a^	c.1A>C	p.Met1?	Start-loss	PM2,PP5, PVS1^d^ (downgraded to Strong)	Likely pathogenic	694505
12	c.5609G>A	p.Arg1870Gln	Missense (Splice region)^e^	PP3,PP4,PP5,PM2,PM5^b^(clinvar:1,070,186 and 1,748,220)	Pathogenic	185354
13	c.616A>T	p.Lys206Ter	Nonsense	PP4,PM2,PVS1	Pathogenic	2443302 - own submission
Heterozygous CNVs identified by MLPA analysis						
Patient	Variant	Nomenclature	Chromosomal Co-ordinates	Size	Interpretation	
14	Type 3 deletion	rsa [GRCh37] 17q11.2(29413855_30348624)x1	chr17:29413855–30348624	∼1Mb	Pathogenic	
15	Two exon deletion	rsa [GRCh37] 17q11.2(29554285_29554561)x1	chr17:29554285–29554561	∼276 bp (exon 19 and exon 20)	Pathogenic	
16	Type 1 deletion	rsa [GRCh37] 17q11.2(29058373_30348624)x1	chr17:29058373–30348624	∼1.4 Mb	Pathogenic	

^a^ Patients 8 and 11 had clinical features of Noonan syndrome and NF1. Other RASopathy genes were screened in these patients but no Noonan syndrome variant was identified.

^b^ PM5 is applied when the variant is observed where another amino acid residue with different missense change was determined as pathogenic.

^c^ PS3 is applied when there’s reliable reference showing that a functional study was done.

^d^ PVS1 code downgraded to a strong code because although the variant is at the initiation codon/start-loss variant the consequence of this variant at a protein level could not be predicted or is unknown (no functional study found).

^e^ Missense (splice region)—missense variant is situated at the splice region (last base pair of coding exon 38).

**TABLE 2:** Comparison of the clinical characteristics noted in Southern African patients with clinically diagnosed NF1, in whom NF1-causing SNV/CNV was (positive cohort) or was not (negative cohort) identified using two-tailed exact Fisher test.

Clinical feature		Number (n/N) (%)		*p*-value^u^ (Fisher’s exact test)
		Positive cohort^a^	Negative cohort^b^	
Demographic information	Age range (months)	6–513	26–401	
	Age median (months)	104	188.5	
	Sex ratio (M:F)	9:6	6:4	
Ectodermal	Café-au-lait macules	14/15 (93)^c^	10/10 (100)^d^	1.00
	Freckling^e^	11/15 (73)	10/10 (100)	0.12
	Cutaneous neurofibromas	10/15 (67)^f^	8/10 (80)^g^	0.66
Ocular	Lisch nodules	2/8 (25)^h^	1/1 (100)^i^	0.33
	Optic gliomas	0/8 (0)^h^	0/1 (0)^i^	1.00
Tumours/malignancies	Plexiform neurofibroma/s	5/15 (33)	4/10 (40)	1.00
	MPNST	0/15 (0)	0/10 (0)	1.00
	Other	3/15 (20)^j^	0/10 (100)	
Neurological	Developmental/intellectual disability	6/15 (40)^k^	4/10 (40)^l^	1.00
	Learning disability	8/15 (53)	5/10 (50)	1.00
	Behavioural problems	1/15 (7)^m^	0/10 (0)	1.00
	FASI	4/7 (57)^n^	0/2 (0)^n^	0.44
	Other	2/15 (13)^o^	1/10 (10)^p^	
Musculoskeletal	Bone dysplasia^q^	0/15 (0)	0/10 (0)	1.00
	Scoliosis^r^	3/15 (20)	2/10 (20)	1.00
CVS/chest	Hypertension	0/15 (0)	0/10 (0)	1.00
Growth centile				
Weight	<3rd	2/13 (15)	1/10 (10)	1.00
	3rd-97th	11/13 (72)	9/10 (90)	1.00
	>97th	0/13 (0)	0/10 (0)	1.00
Length/height	<3rd	3/14 (21)	3/10 (30)	0.67
	<50th	11/14 (79)	10/10 (100)	0.24
	3rd-97th	10/14 (71)^s^	7/10 (70)	1.00
	>97th	1/14 (7)	0/10 (0)	1.00
Head circumference	<3rd	0/15 (0)	1/10 (10)	0.40
	3rd-97th	8/15 (53)^t^	5/10 (50)	1.00
	>50th	14/15 (93)	7/10 (70)	0.27
	>97th	7/15 (47)	4/10 (40)	1.00
Craniofacial features	Dysmorphism	7/15 (47)	4/10 (40)	1.00

Abbreviations: MPNST (malignant peripheral nerve sheath tumour), FASI (focal area of signal intensity), CVS (cardiovascular system).

^a^ Disease-causing SNV/CNV identified.

^b^ No disease-causing SNV/CNV identified in *NF1* or *SPRED1*.

^c^ 13/15 (87%) had more than six café-au-lait macules meeting size criteria for NIH clinical diagnosis of NF1.

^d^ 9/10 (90%) had more than six café-au-lait macules meeting size criteria for NIH clinical diagnosis of NF1.

^e^ Axillary and/or inguinal.

^f^ 8/15 (53%) had more than two cutaneous neurofibromas; 2/15 (13%) had neurofibromas, but the number was not documented.

^g^ 7/10 (70%) had more than two cutaneous neurofibromas.

^h^ 7/15 (47%) had no information available regarding formal ophthalmological assessments.

^i^ 9/10 (90%) had no information available regarding formal ophthalmological assessments.

^j^ Including a cerebellar glioma, a neuroendocrine tumour of the Ampulla of Vater and a brainstem glioma.

^k^ Of varying degrees from mild to moderate; the degree of developmental/intellectual disability was not documented for two patients.

^l^ Of varying degrees from mild to severe.

^m^ Attention deficit hyperactivity disorder.

^n^ 8/15 (53%) of the positive cohort and 8/10 (80%) of the negative cohort had no information available regarding brain imaging.

^o^ Including chronic hydrocephalus, and arachnoid cysts and hydrocephalus.

^p^ West syndrome.

^q^ Long bone or sphenoid wing dysplasia.

^r^ Location and degree not specified.

^s^ 3/14 (21%) had relative short stature.

^t^ 5/15 (33%) had relative macrocephaly.

^u^ Statistically significant if *p*-value < 0.05.

The c.1885G>C (p.Gly629Arg) variant identified in patient 8 was downgraded from likely pathogenic to VUS due to code PS1 being downgraded to moderate as there is no evidence that the c.1885G>C variant (ClinVar ID: 1410579) affects splicing as in the case of c.1885G>A (ClinVar ID: 68308). The codes applicable this variant were, PP4 because the patient met the NHI NF1 criteria, PM2 as the variant was not found in population databases and PS1_moderate which led to VUS classification.

**FIGURE 1:**
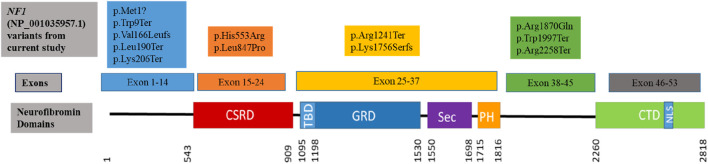
Clustering of the pathogenic and likely pathogenic sequence variants identified in the current study to the neurofibromin domains. (CSRD) Cysteine Serine Rich Domain; (TBD) Tubulin Binding Domain; (GRD) GTPase Activating Protein Related Domain; (SEC) SEC14p Homology Domain; (PH) Pleckstrin Homology Domain; (CTD) C-Terminal Domain; (NLS) Nuclear Localization Site. Variants are coloured to indicate within which exons they occur.

The sequence variants in Table 1 were all previously reported in ClinVar, except for the c.616A>T,p.Lys206Ter variant identified in patient 13, which has not been reported in the literature or ClinVar, suggesting that this variant could be a novel variant. The variant was submitted to ClinVar through current study only (2,443,302). The missense variant c.5609G>A, p.Arg1870Gln identified in patient 12 occurs at last base pair of coding exon 38, which makes it likely to have some effect on normal mRNA splicing. The distribution of the variants throughout the neurofibromin domains is shown in Figure 1, indicating that there is no obvious hotspot region associated with pathogenic variants in the *NF1* gene.

**FIGURE 2:**
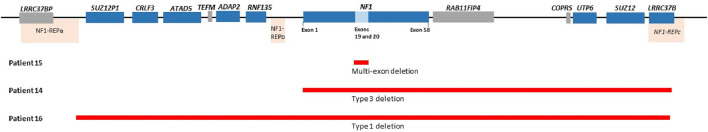
Showing *NF1* gene deletion variants for patient 15 (Exons 19 and 20 deletion), Patient 14 (Type 3 deletion encompassing *NF1* gene extending into other genes downstream of *NF1*) and patient 16 (Type 1 deletion including *NF1* and flanking genes). Please note probe for genes in the blue blocks were included in the MLPA kits used in the current study and the genes in the grey block were not included. Coffalyser ratio charts for these are shown in Supplementary Figure S1.

Two of the three patients with identified CNVs had heterozygous whole gene deletions extending into the genes flanking the *NF1* gene (patient 14 and 16), as shown in Figure 2. The third patient (patient 15) had a deletion of two exons (exon 19 and 20) that has not been reported before in the literature and is shown in Figure 2. Patient 14 had a heterozygous type 3 *NF1* gene deletion encompassing the whole gene and extending into three genes downstream of and flanking *NF1* (*LRRC37B, SUZ12* and *UTP6*). Patient 16 had a type 1 heterozygous gene deletion including the entire *NF1* gene and extending into multiple genes both up- and downstream of and flanking the *NF1* gene (*SUZ12P1, CRLF3, ATAD5, ADAP2, RNF135, UTP6, SUZ12* and *LRRC37B*). Type 1 NF1 deletions typically encompass 1.4 Mb covering the entire *NF1* gene and multiple flanking genes, while type 3 deletions are typically 1 Mb in size. The clinical information for these three patients is shown in Supplementary Table S1 (shaded) and they all met the NIH NF1 diagnostic criteria based on the features they present.

Ten, 10/26 (38%), patients had no variants identified using *NF1* and *SPRED1* SNV analysis or *NF1* CNV analysis. The negative cohort did not have SpliceAI delta scores suggesting high confidence of pathogenicity. No clinically significant variants were identified in other RASopathy genes (*CBL, LZTR1, KRAS, NRAS, PTPN11, RAF1, SOS1, RIT1, SOS2, RASA1, BRAF, MAP2K1, MAP2K2, SPRED1, NF1, NF2, HRAS, SHOC2, RRAS, RASA2, A2ML1*) that were included in the targeted panel (Mudau et al., 2023). In the negative cohort, 3/5 (60%) patients, for whom the information was available, had a documented first degree relative with clinically-diagnosed NF1. Detailed phenotypic information was not available for these relatives.

## Discussion

The study cohort included 26 unrelated patients of Southern African ancestry with clinical features highly suggestive of NF1. A positive detection rate of 58% (15/26) was achieved, with 46% (12/26) being SNVs identified using a NGS targeted gene panel and 12% (3/26) *NF1* deletion variants identified using MLPA CNV analysis. These frequencies are in keeping with known data on NF1 causative variants, with 50%–90% of the variants associated with NF1 identified using sequence analysis and 5%–10% identified using targeted deletion/duplication analysis (Friedman, 1998; Pacot et al., 2021).

Of the 12 pathogenic/likely pathogenic sequence variants identified, ∼92% (11/12) had been previously reported and only one variant, c.616A>T (p.Lys206Ter) identified in patient 11, was novel, not previously reported in disease variant databases or the literature prior to the current study. This nonsense variant substitutes lysine at position 206 introducing a stop codon, resulting in premature termination of translation and a truncated protein. Since this variant is located in exon 6 of 58 exons of the *NF1* gene (Figure 1), premature termination of translation will adversely affect the protein (Svidritskiy et al., 2018). It is expected that the protein will either be absent due to nonsense-mediated decay of the transcripts, or the protein will be significantly disrupted since only a small part will be translated (He and Jacobson, 2015; Kurosaki and Maquat, 2016).

Together with the novel variant, truncating (frameshift and nonsense) variants were identified in 67% (8/12) of the patients; the seven identified variants have all been reported to cause NF1 mostly resulting in haploinsufficiency of the *NF1* gene and loss of function (LoF) of the gene product (Castellanos et al., 2020; Zhang et al., 2021). Missense pathogenic variants altering protein function due to amino acid substitutions accounted for 23% (3/13) of the variants. The high frequency of truncating variants compared to the missense variants was not unique to the present study. In a study by Riva et al., 2022 that reviewed NF1 patient records in Parma hospital in Italy, out of 34 variants identified, 67.6% (23) were frameshift and nonsense compared to one missense variant (Riva et al., 2022). Although there are no significant differences in phenotype severity reported between truncating and missense NF1 variants in the literature as well as the current cohort, a study on NF1 missense variants (Koczkowska et al., 2020) showed that some patients with these variants exhibited some Noonan syndrome phenotypes such as pulmonic stenosis, cardiovascular abnormalities, short stature and macrocephaly. This was, not the case in the three patients (1, 10 and 12) with missense variants in the current cohort, they all met NIH NF1 diagnostic criteria without any apparent overlapping Noonan syndrome features. Missense VUS was identified in patient 8 who met NIH NF1 diagnostic criteria, the variant did not meet the ACMG criteria to be classified as disease causing. A recent study performed in a diagnostic laboratory in Italy, where a 20 years reassessment of *NF1* VUSs, 85 out of 589 (14%) NF1 tests requested. Of these VUSs 66% of missense VUSs were reclassified to likely pathogenic/pathogenic (Martorana et al., 2023). It is likely that the VUS identified in the current study will be reassessed in the future when more functional evidence is available.

The pathogenic variants identified in this study are distributed throughout the *NF1* gene as seen in Figure 1. Five variants (all previously reported to be associated with NF1) were clustered in the region upstream of the CSRD domain. Although this region of the gene is uncharacterised and not within any known functional domain, it appears to be a critical region for truncating NF1-causing variants as reported in this study, as well as various other studies (Sabbagh et al., 2013; Abdel-Aziz et al., 2021). Three variants, two of which were protein truncating variants, clustered upstream of the CTD domain which is responsible for regulating the neurofibromin phosphorylation activity that promote cell proliferation (Bergoug et al., 2020). Four variants were within the CSRD, GRD and PH functional neurofibromin domains. Variants in these protein domains are reported to affect the protein activity, leading to loss of function of the neurofibromin protein due to haploinsufficiency (Bergoug et al., 2020; Zhang et al., 2021). The distribution of the variants in the *NF1* gene protein domains is similar to the one observed in the literature where distribution of various pathogenic variant types throughout the gene with no obvious hotspot region (Friedman, 1993b; Abdel-Aziz et al., 2021; Legius et al., 2021; Riva et al., 2022). Missense variants were observed to cluster in the CSRD domain (Figure 1) with no truncating variant observed, however the sample size in the current study is too small to make significant discussion on these.

Of the three patients with NF1 CNVs, a type 1 heterozygous deletion was identified in one patient (patient 16). This type of a deletion is the most commonly identified large deletion in NF1 patients and is typically ∼1.4 Mb in size and has defined recurrent breakpoints *NF1*-REPa and *NF1*-REPc (Pasmant et al., 2010; Pacot et al., 2021; Kehrer-Sawatzki and Cooper, 2022). A second, type 3 heterozygous deletion identified in patient 14 is a rarer deletion reported to be ∼1 Mb in size (Kehrer-Sawatzki and Cooper, 2021; 2022). A novel heterozygous two-exon deletion was identified in patient 15 and has not been reported in CNV databases such as DECIPHER. This deletion was reported as clinically significant due to haploinsufficiency secondary to protein truncation.

Overall, the clinical features identified in the present study group were consistent with the known phenotype associated with NF1. Of the positive cohort, most of the patients [87% (13/15)] met the NIH clinical diagnostic criteria for NF1 (Legius et al., 2021). The two remaining patients (patient 9 and patient 11), who did not meet these criteria, were included in the study on the basis of supportive features; patient 9 (with c.5991G>A, p.Trp1997Ter pathogenic variant) had a FASI identified on MRI brain scan and patient 11 (with c.1A>C, p.Met1? Likely pathogenic variant) had an incomplete assessment which was lacking a formal ophthalmological examination but had other suggestive features, with some suggestive of a NF1-Noonan syndrome (NS) phenotype (NFNS, OMIM# 601321); the c.1A>C (p.Met1?) likely pathogenic variant identified in this patient has one submission on ClinVar (in addition to the one identified in the current study). This submission (ClinVar ID, 694,505) was reported in a patient with NF1, this suggests that although the patient was reported to have a NFNS phenotype, the *NF1* variant could be responsible for the clinical presentation. A NFNS phenotype is reported to occur in approximately 12% of cases of NF1 (Friedman, 1993a; Stevenson et al., 2006). Two patients, patient 8 with the *NF1* VUS and patient 11, were suspected as having a NFNS phenotype. Although the diagnosis of NF1 in patient 8 has not yet been molecularly confirmed, this patient meets NIH criteria for NF1 and, together with patient 11, would result in a NFNS phenotype frequency of 13% in this cohort, in keeping with the reported frequency of NFNS phenotypes. Patient 8 and 11 also had full RASopathy panel screening and were found to not have any other variant except the variant identified in the *NF1* gene. The start-loss variant identified in patient 11 (c.1A>C, p.Met1Leu) may need additional information to confirm pathogenicity since variants occurring in the first codon of a gene needs additional transcript information or functional evidence to interpret (Abou Tayoun et al., 2018). Similarly the variant identified in patient 8 (c.1885G>C,p.Gly629Arg) was classified as VUS due to the need to downgrade code PS1 that was applied because another variant c.1885G>A (VCV000068308.47) with the same amino acid change was classified as pathogenic. However there is a functional study supporting the effect of c.1885G>A variant on the protein (Pros et al., 2008) and there is no study on the effect of c.1885G>C variant on the protein function leading to classification of VUS (Richards et al., 2015).

Individuals with NF1 typically have an above-average head circumference with only a minority having a head circumference that is greater than 4 SD above the mean for age (Friedman, 1993b). Of the total positive cohort, 93% (14/15) had an above-average head circumference and 80% (12/15), in whom the information was available, had either a documented macrocephaly or relative macrocephaly. Only one patient [7% (1/15)] had a head circumference that was greater than four SD above the mean for age. Of the SNV-positive patients the majority [75% (9/12)], for whom information was available, had either macrocephaly or relative macrocephaly. These findings are all in keeping with the known NF1 phenotype. Macrocephaly is reported to correlate with optic pathway gliomas in approximately 62% of children with NF1 (Schindera et al., 2011); however there were no documented optic gliomas (past or present) noted in any of the present study’s positive patients with true macrocephaly, in whom an ophthalmology assessment had been performed.

Individuals with NF1 typically also have a below-average height with only a minority having a height that is greater than three SD below the mean for age. In the present study, below-average height was documented in 79% (11/14) of the total positive cohort, for whom data was available. This finding is consistent with the known height phenotype of NF1. Only 3/14 (21%) in whom the information was available, and all with a pathogenic SNV identified, had short stature with a height below the third centile and greater than two SD below the mean for age. Only one patient had a height that was greater than three SD below the mean for age.

The frequency of short stature in children with NF1 varies across the literature from 8% to 33%, with the frequency of short stature in the present study’s cohort falling within this range and being a similar frequency (27%) to that reported by North. (1993) (North, 1993; Zessis et al., 2018).

Of the positive cohort, only 33% (5/15) had a documented plexiform neurofibroma/s, a lower frequency than the reported frequency of approximately 50% in the literature (Friedman, 1993a; Legius et al., 2021). This is likely because many plexiform neurofibromas are located deep in the body and are typically only identified on detailed radiological imaging, which was lacking and was not performed routinely in our cohort. In the State healthcare sector in South Africa, and possibly in other low- or middle-income countries, patients with NF1 are not routinely investigated for plexiform neurofibromas unless there are signs and/or symptoms to suggest the presence of one. The lower frequency of plexiform neurofibromas in the present study may also be influenced by age-related penetrance, as the present positive cohort was predominantly paediatric in age. One-third of the SNV-positive cohort 33% (4/12) had one or more plexiform neurofibroma. Patient 6, identified to have a frameshift variant (c.5267_5268del), had a higher burden of plexiform neurofibromas (three in total) and was also found to have a brainstem glioma.

Intellectual disability, learning difficulties and behavioural problems are all known to occur in patients with NF1 (Sabbagh et al., 2013; Mao et al., 2018; Kehrer-Sawatzki and Cooper, 2022). A high frequency [40% (6/15)] of the present positive cohort had documented developmental or intellectual disability, a much higher frequency than that of between 4 and 8% noted in a review of the literature (Vogel et al., 2017). The high frequency noted in the present study is likely an overestimate due to possible ascertainment bias, it is suspected that individuals with NF1 who have developmental or intellectual disability are more likely to be referred to our local genetic clinics. It must be noted that a formal neurodevelopmental assessment was not part of this study and these diagnoses were based on clinical history and hospital file notes.

Among other features, the NF1 microdeletion phenotype is associated with somatic overgrowth, dysmorphic features (notable in adolescence and adulthood), more severe cognitive challenges, the earlier appearance with greater numbers of neurofibromas, and an increased risk of developing MPNST (Pasmant et al., 2010). Of the present study’s CNV-positive cohort, 67% (2/3) in the present study had macrocephaly, 33% (1/3) had relative macrocephaly, 100% (3/3) had normal weight, and none of the patients had tall stature. None of the deletion-positive patients were noted to have significant facial dysmorphism, however, these patients were relatively young and facial dysmorphism may still develop over time. All three deletion-positive patients had more than two cutaneous neurofibromas, with patient 15 also having a plexiform neurofibroma; however, the exact number and ages at which the neurofibromas developed were not documented in the clinical notes. There were no documented histories of MPNST in any of the CNV-positive patients. Intellectual disability is also well described in the NF1 microdeletion phenotype and occurs in over half of patients with the NF1 microdeletion phenotype (Legius et al., 2021; Kehrer-Sawatzki and Cooper, 2021). Although a small sample size, this was consistent with the present study’s deletion-positive cohort where 67% (2/3) had documented developmental/intellectual disability, and all three patients [100% (3/3)] had a documented learning disability. Again, it must be noted that a formal neurodevelopmental assessment was not part of this study and these diagnoses were based on clinical history and hospital file notes. Prospective studies on a larger sample size of South African patients are needed to adequately comment on the deletion-phenotype of NF1 in Southern African patients.

Of the present study cohort, 38% (10/26) tested negative for a disease-causing SNV/CNV. All 10 patients within the present negative-cohort met NIH clinical diagnostic criteria for NF1 (Legius et al., 2021). There were no significant differences observed in the clinical characteristics between the negative and positive cohort (Table 2).

The cross-sectional nature of the clinical data collection would also have influenced the frequencies of clinical features reported, and therefore a more prospective study on the clinical features and genotype/phenotype correlations in a larger cohort of Southern African patients with NF1 would be valuable. Of concern is that the less complicated cases of NF1 are perhaps not being referred to the genetic services, and therefore may not be receiving the necessary genetic counselling and medical surveillance that they require. Of special mention, only 35% (9/26) of the entire study cohort had undergone a formal ophthalmological assessment. Although NF1 is a relatively common genetic condition which presents to various other clinics (e.g., paediatric, adult neurology, oncology), based on the data obtained from clinical notes it appears that the clinical workup of a suspected NF1 patient and medical surveillance of a clinically diagnosed patient with NF1 is often incomplete in our local clinical setting. Patients with NF1 are not always routinely referred to, or do not always have easy access to, the genetic services in South Africa. It is possible that the more ‘obvious’ or apparently severe cases of NF1 are referred more readily to the genetic clinic and therefore there may be an element of ascertainment bias.

Although NF1 can be clinically diagnosed in most cases, genetic testing has a role to play in certain instances. These include confirming cases that do not strictly meet NIH NF1 criteria, informing clinical surveillance, and allowing for cascade and prenatal testing. Patients with NF1 often present in infancy or childhood and a confirmed genetic diagnosis would enable early diagnosis and implementation of formal medical surveillance from a young age, thereby potentially reducing the morbidity and mortality associated with NF1-complications. Genetic diagnosis may also allow for genotype-phenotype correlation with improved counselling and management, as well as potentially enabling patients to take part in future trials for molecular-targeted therapies. We would recommend that all South African State healthcare patients suspected of having NF1, regardless of perceived severity, be referred to the genetic services available in city centres across South Africa. Genetic services can provide genetic counselling, clinical assessment, implementation of appropriate medical surveillance, identification of other at-risk family members, and diagnostic and/or prenatal genetic testing. Currently these services are available in three provinces Gauteng, Western Cape and Free State in South Africa with patients from other rural provinces having to travel to these three for mostly clinical phenotyping and genetic testing only offered when indicated.

## Conclusion

This is the first study documenting both the molecular and clinical characteristics of NF1 in a group of Southern African patients. This study was able to molecularly confirm the diagnosis of NF1 in 58% of the study group. Of the 15 positive variants identified, two variants (c.616A>T and the exon 19 and 20 deletion) have not been reported previously and may be novel variants. The majority of our patients met NIH clinical diagnostic criteria for NF1, yet 38% had no identifiable disease-causing SNV or CNV in *NF1*. These patients could benefit from future testing using *SPRED1* CNV analysis, *NF1* RNASeq or full gene sequencing to detect splice-site variants and other complex variants, however some of these may be somatic mosaic. Screening somatic mutations in the neurofibroma tissues may also identify mutations causing NF1 in these patients. Our study also confirms that the clinical features of Southern African patients with NF1 are largely similar to that of the known NF1 phenotype, with the exception of a lower frequency of plexiform neurofibromas and a higher frequency of developmental/intellectual disability compared to other cohorts, possibly reflecting ascertainment bias. In the State healthcare setting in South Africa, it is recommended that all patients suspected of having NF1 be referred to genetic services in order to access genetic counselling and testing, as well as guidance related to the recommended medical surveillance. Prior to the current study, these patients did not have a molecular confirmation for their clinical diagnosis. The identification of a causative variant is beneficial in interventions such as tumour surveillance as well as inclusion in NF1 clinical trials This study shows that by utilizing an NGS targeted panel, followed by MLPA CNV analysis, many patients can now obtain a molecular diagnosis which was not available previously.

## Data Availability

The datasets presented in this study can be found in online repositories. The names of the repository/repositories and accession number(s) can be found below: https://www.ncbi.nlm.nih.gov/clinvar/submitters/508172/, ClinVar -Organization ID 508172.
