# Supplementary material for: Mutation analysis and clinical profile of South African patients with Neurofibromatosis type 1 (NF1) phenotype
Source: Front Genet. 2024 Mar 26;15:1331278. doi: 10.3389/fgene.2024.1331278 (PMC11002079; doi:10.3389/fgene.2024.1331278)
Supplement: Supplementary file 1 [file Table1.docx]

**Supplementary tables**

**Table S1:** Demographic features, clinical characteristics and molecular findings of 15 Southern African patients, with clinically diagnosed NF1, with a disease-causing variant identified in the *NF1* gene.

| **Clinical features** | **Patient number** | | | | | | | | | | | | | | |
| --- | --- | --- | --- | --- | --- | --- | --- | --- | --- | --- | --- | --- | --- | --- | --- |
|  | **1** | **2** | **3** | **4** | **5** | **6** | **7** | **9** | **10** | **11^a^** | **12** | **13** | **14** | **15** | **16** |
| **Demographic information** |  |  |  |  |  |  |  |  |  |  |  |  |  |  |  |
| Sex | F | F | M | F | M | M | M | M | F | M | M | F | M | M | F |
| Age at recruitment | 1y11m | 13y11m | 8y8m | 6y6m | 28y0m | 13y4m | 4y10m | 9y9m | 42y9m | 5y8m | 0y6m | 2y4m | 6y6m | 12y3m | 12y2m |
| Ethnolinguistic group | Zulu/ Seswati | NA | Zulu | NA | Shona | Sotho/ Venda | NA | Tswana | Tswana | NA | NA | Sotho | Xhosa | NA | Zulu |
| **NIH NF1 criteria met** | + | + | + | + | + | + | + | - | + | - | + | + | + | + | + |
| **Growth centiles (SDS)** |  |  |  |  |  |  |  |  |  |  |  |  |  |  |  |
| Weight | 3rd-10th (-1.86) | 10th-25th (-0.78) | 10th-25th (-0.97) | NA | NA | 90th-97th (1.47) | 10th-25th (-1.17) | 10th (-1.56) | 50th (-0.29) | <3rd (-2.05) | <3rd (-2.36) | 25th (-0.53) | 50th (0.20) | 10th-25th (-1.19) | 50th-75th (0.25) |
| Length/height | <3rd (-2.14) | 3rd-10th (-1.67) | 25th-50th (0.27) | 50th-75th (0.30) | NA | >97th (>3.00) | 3rd-10th (-1.62) | 10th (-1.92) | <3rd (-2.75) | 3rd-10th (-1.44) | <3rd (>-3) | 3rd-25th (-1.33) | 25th-50th (-0.29) | 10th-25th (-1.23) | 90th-97th (1.49) |
| Head circumference | >97th (2.84) | >97th (>4) | >97th (2.64) | 75th (1.17) | 90th-97th (1.36) | >97th (3.70) | 75th-90th (1.15) | 10th-25th (-1.00) | 75th (1.18) | 50th-75th (0.54) | 75th-90th (0.55) | >97th (2.08) | >97th (2.29) | 50th-75th (0.00) | >97th (3.84) |
| **Ectodermal** |  |  |  |  |  |  |  |  |  |  |  |  |  |  |  |
| CAL (number) | + (>6) | + (>6) | + (>6) | + (>6) | + (>6) | + (>6) | + (>6) | + (3) | - | + (>6) | + (>6) | + (>6) | + (>6) | + (>6) | + (>6) |
| Axillary/inguinal freckling | + | + | + | + | + | + | - | + | - | - | + | - | + | + | + |
| Cutaneous neurofibromas (number) | - | + (>2) | + (>2) | + | + (>2) | + | + (>2) | - | + (>2) | - | - | - | + (>2) | + (>2) | + (>2) |
| Other | Hyperkeratosis, dry skin | - | - | Deep palmar creases | - | - | Hypopigmentation | - | - | - | - | - | - | - | Hyperpigmented patches |
| **Ocular** |  |  |  |  |  |  |  |  |  |  |  |  |  |  |  |
| Lisch nodules (number) | - | - | - | NA | NA | - | NA | - | NA | NA | NA | + (NA) | - | + (NA) | NA |
| Optic glioma (number) | - | - | - | NA | NA | - | NA | - | NA | NA | NA | - | - | - | NA |
| **Tumours/ malignancies** |  |  |  |  |  |  |  |  |  |  |  |  |  |  |  |
| Plexiform neurofibroma (number) | - | + (1) | - | + (1) | + (1) | + (3) | - | - | - | - | - | - | - | + (1) | - |
| MPNST | - | - | - | - | - | - | - | - | - | - | - | - | - | - | - |
| Other | - | Cerebellar glioma | - | - | Ampulla of Vater neuroendocrine tumour | Brainstem glioma | - | - | - | - | - | - | - | - | - |
| **Neurological** |  |  |  |  |  |  |  |  |  |  |  |  |  |  |  |
| Headaches/ migraines | - | + | - | - | - | - | - | - | - | - | - | - | - | + | - |
| Developmental/ intellectual delay (degree) | + (mild) | + (mod) | - | - | - | - | - | + (NA) | - | + (mild to mod) | - | - | + (NA) | - | + (mild) |
| Learning disability | + | + | - | - | - | + | - | + | - | + | - | - | + | + | + |
| Behavioural problems | - | - | - | - | - | - | - | + (ADHD) | - | - | - | - | - | - | - |
| MRI brain scan | + | + | NA | NA | NA | + | NA | + | NA | NA | + | + | NA | + | NA |
| FASI | - | - | NA | NA | NA | + | NA | + | NA | NA | - | + | NA | + | NA |
| Other | - | Chronic hydrocephalus | - | - | - | - | - | - | - | - | - | - | Arachnoid cyst, hydrocephalus | - | - |
| **Musculoskeletal** |  |  |  |  |  |  |  |  |  |  |  |  |  |  |  |
| Long bone or sphenoid wing dysplasia | - | - | - | - | - | - | - | - | - | - | - | - | - | - | - |
| Scoliosis | - | + | - | - | - | + | - | - | - | - | - | - | - | - | + |
| Other | Pectus carinatum, joint hyperlaxity | - | - | Short 5th metacarpal | - | - | - | - | - | Clinodactyly | - | Genu valgum | - | - | Genu valgum, overlapping toes |
| **CVS/chest** |  |  |  |  |  |  |  |  |  |  |  |  |  |  |  |
| Hypertension | - | - | - | - | - | - | - | - | - | - | - | - | - | - | - |
| History of CVA | - | - | - | - | - | - | - | - | - | - | - | - | - | - | - |
| Other | - | - | - | - | - | - | - | - | - | Broad chest, widely spaced nipples | - | - | - | - | - |
| **Craniofacial** |  |  |  |  |  |  |  |  |  |  |  |  |  |  |  |
| Ocular hypertelorism | - | - | + | - | - | - | - | - | - | + | - | - | - | - | + |
| DSPF | + | - | + | + | - | - | - | - | - | + | - | - | - | - | - |
| Low-set ears | + | - | - | - | - | - | - | - | - | + | - | - | - | - | - |
| Webbed neck | - | - | - | - | - | - | - | - | - | + | - | - | - | - | - |
| Other | Frontal bossing, full lips, high-arched palate, Widow’s peak | - | Epicanthic folds | Laterally sparse eyebrows, broad nasal tip, short philtrum | Mildly coarse face | - | - | - | Long face, malar hypoplasia | Ptosis, micrognathia | - | - | Micrognathia, full upper lip | - | Broad nasal root |
| **FDR meeting NHI NF1 criteria** | - | - | + | - | - | - | - | - | + | - | + | - | NA | - | - |
| **Clinically significant variant identified**  ***NF1* gene: NM_001042492.3** | c.1658A>G (p.His553Arg) | c.6772C>T (p.Arg2258Ter) | c.496_497del (p.Val166Leufs) | c.3721C>T (p.Arg1241Ter) | c.569T>G p.Leu190Ter | c.5267_5268del (p.Lys1756Serfs) | c.27G>A (p.Trp9Ter) | c.5991G>A (p.Trp1997Ter) | c.2540T>C (p.Leu847Pro) | c.1A>C (p.Met1?) | c.5609G>A (p.Arg1870Gln) | c.616A>T (p.Lys206Ter) | Heterozygous *NF1* gene deletion (Type 3) | Exon 19 and 20 deletion | Heterozygous *NF1* gene deletion and flanking genes (Type 1) |
| Type | Missense | Nonsense | Frame-shift | Nonsense | Nonsense | Frame-shift | Nonsense | Nonsense | Missense | Start-loss | Missense | Nonsense | Type 3 deletion | Two exon deletion | Type 1 deletion |
| Interpretation | Pathogenic | Pathogenic | Pathogenic | Pathogenic | Pathogenic | Pathogenic | Pathogenic | Pathogenic | Pathogenic | Likely Pathogenic | Pathogenic | Pathogenic | Pathogenic | Pathogenic | :Pathogenic |
| ClinVar ID | 420076 | 230389 | 431562 | 361 | 431974 | 1072614 | 1459748 | 233869 | 68323 | 694505 | 185354 | 2443302* | NA | NA | NA |

**Abbreviations**

NF1 (neurofibromatosis type 1), F (female), M (male), y (year), m (month), NA (not available), NIH (National Institutes of Health), NF1 (neurofibromatosis type 1), + (yes/present), - (no/absent), SDS (standard deviation score), CAL (café-au-lait macule), MPNST (malignant peripheral nerve sheath tumour), mod (moderate), ADHD (attention deficit hyperactivity disorder), MRI (magnetic resonance imaging), FASI (focal area of signal intensity), CVS (cardiovascular system), CVA (cerebral vascular accident), DSPF (down slanted palpebral fissures), FDR (first degree relative).

**Key**

CNV positive cases are shaded in grey. Absent/unavailable clinical information is indicated by NA (not available).^a^ Patient 11 was noted to have a Noonan syndrome / NF1 phenotype. ClinVar ID (*) identified only in the current study and submitted to ClinVar. Patients shaded in grey were found to have deletion variants; these do not have ClinVar IDs, as the exact variation could not be determined using MLPA analysis.

**Table S2:** Demographic features, clinical characteristics and molecular results of 10 Southern African patients, with clinically diagnosed NF1, with no identified disease-causing SNV/CNV in *NF1* or *SPRED1*.

| **Clinical features** | **Patient number** | | | | | | | | | |
| --- | --- | --- | --- | --- | --- | --- | --- | --- | --- | --- |
|  | 17 | 18 | 19 | 20 | 21 | 22 | 23 | 24 | 25 | 26 |
| **Demographic information** |  |  |  |  |  |  |  |  |  |  |
| Sex | M | M | F | M | M | M | M | F | F | F |
| Age | 12y8m | 33y5m | 7y3m | 2y2m | 6y7m | 27y6m | 18y9m | 6y8m | 29y8m | 33y |
| Ancestry | African | African | African | African | African | African | African | African | African | African |
| Ethnolinguistic group | Sesotho | Zulu | NA | NA | NA | NA | NA | NA | Sesotho | NA |
| **NIH NF1 criteria met** | + | + | + | + | + | + | + | + | + | + |
| **Growth centiles (SDS)** |  |  |  |  |  |  |  |  |  |  |
| Weight | 10th-25th (-0.87) | 3rd-97th (-0.82) | 10th-25th (-1.19) | 25th-50th (0.00) | 10th-25th (-1.13) | 3rd-97th (-0.80) | <3rd (-2.54) | 3rd-97th (+0.96) | 3rd-97th (+0.17) | 25th (-0.88) |
| Length/height | 10th-25th (-1.17) | <3rd (-2.17) | 10th (-1.18) | 25th-50th (-0.28) | 25th-50th (-1.15) | <3rd (-2.24) | 10th-25th (-1.13) | 3rd-97th (-0.29) | <3rd (-2.21) | 10-25th (-1.84) |
| Head circumference | >97th (+2.00) | >97th (>+3.00) | 50th-75th (+0.33) | <3rd (-2.53) | 25th-50th (-0.31) | >97th (+2.46) | 90th-97th (+1.36) | >97th (+3.62) | 3rd-97th (-0.36) | 90-97th (1.18) |
| **Ectodermal** |  |  |  |  |  |  |  |  |  |  |
| CAL (number) | + (>6 ) | + (>6) | + (>6) | + (>6) | + (>6) | + (>6) | + (>6) | + (>6) | + (1) | +(>6) |
| Axillary/inguinal freckling | + | + | + | + | + | + | + | + | + | + |
| Cutaneous neurofibromas (number) | + (>2) | + (>2) | + (>2) | + (1) | + (>2) | + (>2) | - | - | + (>2) | + (many) |
| Other | - | - | - | Velvety skin | Velvety skin, deep palmar creases | Melanocytic naevi | Hyperkeratosis, coarse hair | - | - | - |
| **Ocular** |  |  |  |  |  |  |  |  |  |  |
| Lisch nodules (number) | + (NA) | NA | NA | NA | NA | NA | NA | NA | NA | NA |
| Optic glioma (number) | - | NA | NA | NA | NA | NA | NA | NA | NA | NA |
| **Tumours/malignancies** |  |  |  |  |  |  |  |  |  |  |
| Plexiform neurofibroma (number) | - | + (2) | - | - | - | + (1) | - | + (1) | - | + (1) |
| MPNST | - | - | - | - | - | - | - | - | - | - |
| **Neurological** |  |  |  |  |  |  |  |  |  |  |
| Headaches/ migraines | + | - | - | - | - | - | - | - | - | - |
| Developmental/ intellectual delay (degree) | - | - | - | + (severe) | + (mild to mod) | + (mild) | + (mod) | - | - | - |
| Learning disability | - | + | - | + | + | + | + | - | - | - |
| Behavioural problems | - | - | - | - | - | - | - | - | - | - |
| MRI brain scan | + | + | NA | NA | NA | NA | NA | NA | NA | NA |
| FASI | - | - | NA | NA | NA | NA | NA | NA | NA | NA |
| Other | - | - | - | West syndrome | - | - | - | - | - | - |
| **Musculoskeletal** |  |  |  |  |  |  |  |  |  |  |
| Long bone or sphenoid wing dysplasia | - | - | - | - | - | - | - | - | - | - |
| Scoliosis | + | + | - | - | - | - | - | - | - | - |
| Other | Pectus excavatum, hemihypertrophy, joint hyperlaxity | - | - | - | - | - | - | Pectus excavatum | - | - |
| **CVS/ chest** |  |  |  |  |  |  |  |  |  |  |
| Hypertension | - | - | - | - | - | - | - | - | - | - |
| History of CVA | - | - | - | - | - | - | - | - | - | - |
| Other | - | - | - | Congenital cardiac lesion (not specified) | - | - | - | - | - | - |
| **Craniofacial** |  |  |  |  |  |  |  |  |  |  |
| Ocular hypertelorism | + | - | + | - | - | - | + | + | - | - |
| DSPF | - | - | - | + | + | - | - | - | - | - |
| Low-set ears | + | - | - | - | + | - | - | - | - | - |
| Webbed neck | - | - | - | - | - | - | - | - | - | - |
| Other | Upslanted PFs, high arched palate, overfolded ear helices | - | - | - | Ptosis, long PFs, large mouth, full lips, fleshy ears | Gum hypertrophy | Coarse face, triangular face, prominent chin | - | - | - |
| **FDR meeting NHI NF1 criteria** | - | + | NA | NA | NA | NA | NA | + | - | + |

**Abbreviations**

NF1 (neurofibromatosis type 1), SNV (single nucleotide variant), CNV (copy number variant), M (male), F (female), y (years), m (months), NA (not available), NIH (National Institutes of Health), NF1 (neurofibromatosis type 1), + (yes/present), - (no/absent), SDS (standard deviation scores), CAL (café-au-lait macule), MPNST (malignant peripheral nerve sheath tumour), mod (moderate), MRI (magnetic resonance imaging), FASI (focal area of signal intensity), CVS (cardiovascular system), CVA (cerebral vascular accident), DSPF (down slanted palpebral fissures), PFs (palpebral fissures), FDR (first degree relative).

a)


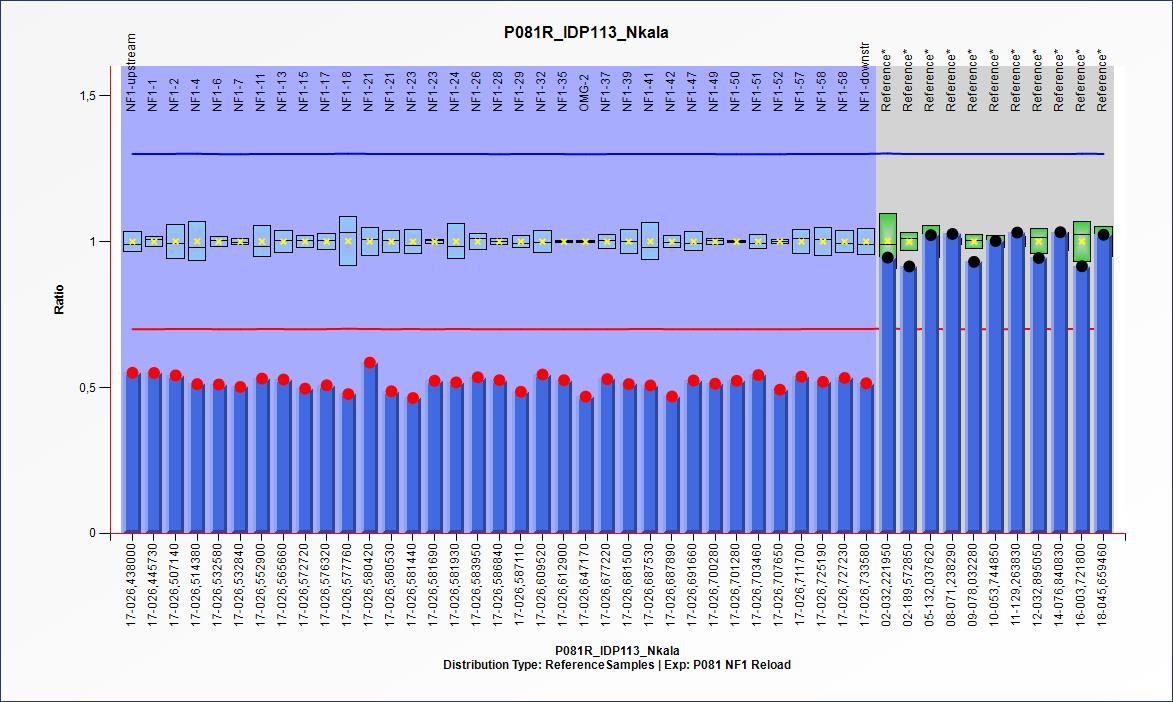

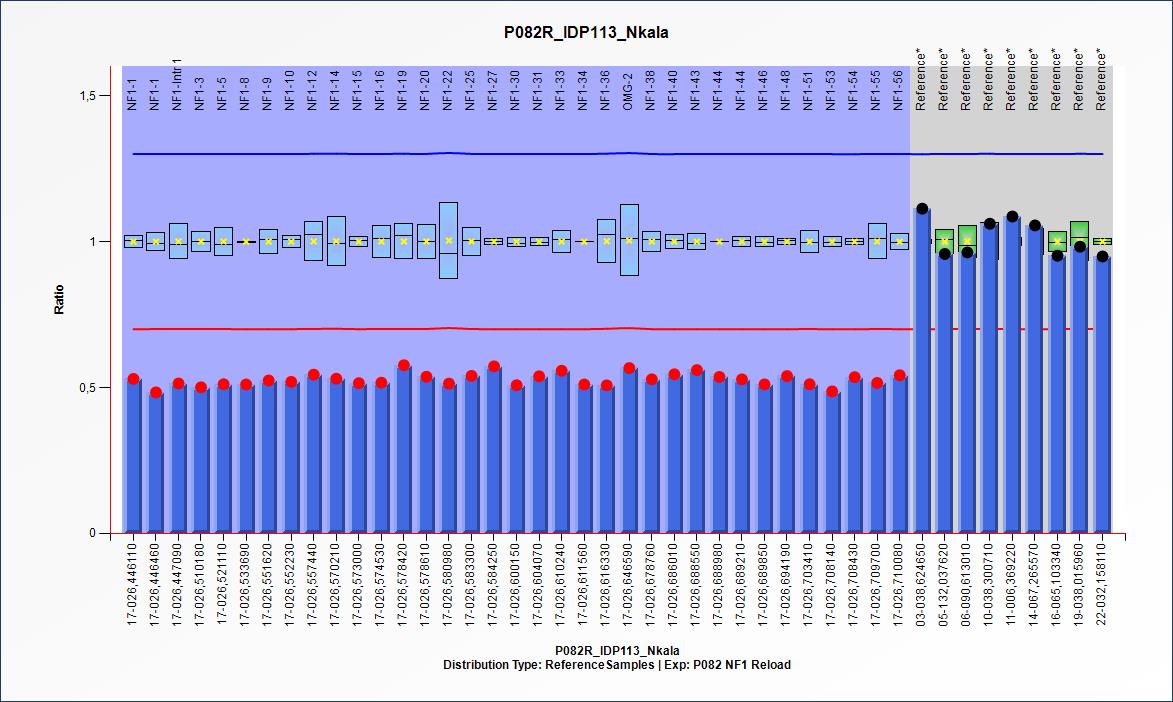

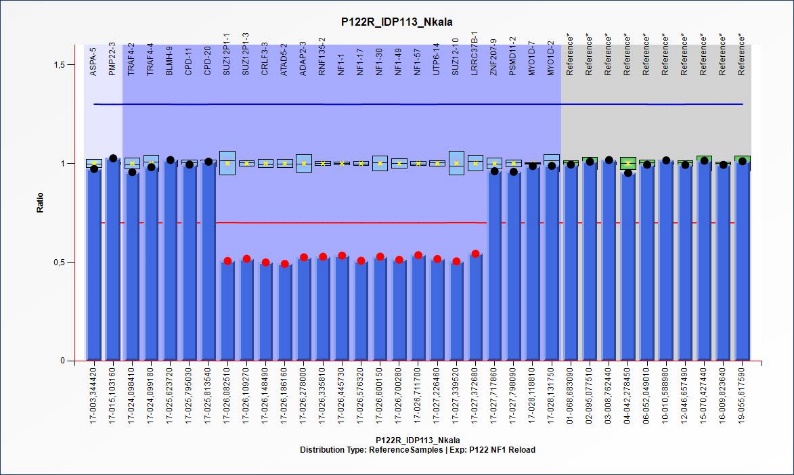


b)

**c)**

**Figure S1**: Type 1 deletion shown in Patient 16; full *NF1* gene deletion with extension to the genes flanking the *NF1* gene (S*UZ12P1, CRLF3, ATAD5, ADAP2* and *RNF135* upstream); (*LRRC37B, SUZ12* and *UTP6 downstream*) identified using MLPA probe mix P081 a), P082 b) and P122 c).
